# Operationalizing focus-sensitivity in a cross-linguistic context

**DOI:** 10.1007/s11050-025-09243-w

**Published:** 2025-12-09

**Authors:** Deniz Özyıldız, Ciyang Qing, Floris Roelofsen, Wataru Uegaki, Maribel Romero

**Affiliations:** 1 Institute for Linguistics, University of Stuttgart, Keplerstr. 17, D-70174 Stuttgart, Germany; 2 Linguistics and English Language, School of Philosophy, Psychology and Language Sciences, University of Edinburgh, Dugald Stewart Building, 3 Charles Street, Edinburgh, EH8 9AD UK; 3 Institute for Logic, Language and Computation (ILLC), University of Amsterdam, P.O. Box 94242, 1090 GE Amsterdam, The Netherlands; 4 Department of Linguistics, University of Konstanz, Room G222, Fach 181, Universitaetsstr. 10, D-78464 Konstanz, Germany

**Keywords:** Focus-sensitivity, Attitude predicates, Clause-embedding predicates, Cross-linguistic data collection methodology

## Abstract

This paper evaluates empirical methods for detecting the focus-sensitivity of clause-embedding predicates, with the objective of making them applicable to predicates within and across languages. Our positive proposal is a test based on a combination of inferential judgments and truth-value judgments. We compare this method with three alternatives: one based on truth-value judgments alone; another based on judgments of coherence or contradiction; and a third one based on inferential judgments alone. We conclude that our combined test is more successful than its alternatives in terms of cross-linguistic uniformity (i.e., it is applicable to different languages with as few language-specific elements involved as possible) and item generalizability (i.e., there is a clear recipe for how to carry out the test for any clause-embedding predicate).

## Introduction

The observation that the semantics of some (but not all) clause-embedding predicates is focus-sensitive, i.e., that these are sensitive to the focus structure of their complements, dates back at least to Dretske (1972).^1^ An example of a focus-sensitive predicate in English is *hope*, whose focus-sensitivity can be observed in a minimal pair such as the following:

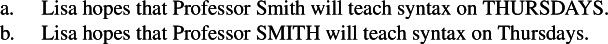

These two sentences are different only in the location of focus within the complement clause. Yet, their truth conditions are distinct, as one can construct a situation in which one is true and the other is false. For example, if Lisa’s only preference is that syntax should be taught on Thursdays and she does not care about which professor will teach it, (1a) is true but (1b) is not.

A number of analyses of the syntactic and semantic behaviours of clause-embedding predicates make crucial use of the property of focus-sensitivity. This includes, among others, Villalta’s (2008) analysis of mood licensing, Romero’s (2015) analysis of the selectional restrictions of emotive factives, Uegaki and Sudo’s (2019) analysis of the selectional restrictions of non-veridical preferential predicates, and Wehbe and Flor’s (2022) analysis of homogeneity effects in clausal complements. In order to evaluate such analyses across predicates and across languages, it is crucial that researchers have a reliable empirical method for detecting the focus-sensitivity of a given clause-embedding predicate. The goal of this paper is to provide a critical assessment of the available methods for detecting focus-sensitivity and to provide a positive recommendation for a method that combines an inferential task and a truth-value judgment task.

To assess these, we will follow Tonhauser and Matthewson (2016) in establishing desiderata for semantic elicitation methods. Tonhauser and Matthewson list **stability**, **replicability** and **transparency** as desiderata that any semantic elicitation method should adhere to. (The definitions of these desiderata will be clarified in Sect. 3.2.) In addition to these general desiderata, we highlight **cross-linguistic uniformity** and **item generalizability** as additional considerations relevant for our evaluation of different tests for focus-sensitivity. In other words, we consider a method preferable if it can be applied with minimal reliance on language-specific elements (cross-linguistic uniformity) and offers a clear, generalizable procedure for applying the task to any item, in our case, clause-embedding predicates (item generalizability).

These desiderata will guide us in the comparison of the merits of different methods for detecting focus-sensitivity, to be detailed in Sects. 4 through 7. In particular, we will claim that existing methods that rely only on truth-value judgments or judgments about coherence/contradiction will fail to satisfy **cross-linguistic uniformity** and/or **item generalizability**. A method based solely on truth-value judgments (Villalta 2008; Harner 2016) fails **item generalizability** in that there is no general procedure for generating the context against which the truth values of the relevant test sentences should be judged. A method based on coherence/contradiction (Villalta 2008, pp. 497–498) turns out to fail **cross-linguistic uniformity** due to the presence of language-specific expressions of denial in the example dialogues to be tested. In contrast, the method we will favor, i.e., the method that combines inferential judgments and truth-value judgments, will turn out to satisfy both **cross-linguistic uniformity** and **item generalizability**. We will also argue that the combined method is no worse than its alternatives in terms of the other general desiderata—**stability**, **replicability** and **transparency**.

A more general aim of the paper is to contribute to the ongoing discussion on the methodology of controlled semantic data collection in the cross-linguistic context (Matthewson 2004; Tonhauser and Matthewson 2016) by examining the methods for testing focus-sensitivity. Tonhauser and Matthewson (2016, p. 3) invite the semantics community to a “collaborative process of developing consistent standards for empirical evidence about meaning”. In this paper, we further this discussion by highlighting specific desiderata—i.e., **cross-linguistic uniformity** and **item generalizability**—in the context of cross-linguistic investigation, using focus-sensitivity as a case study. Rather than promoting a single optimal method, our aim is to critically evaluate the range of available approaches, clarify their respective trade-offs, and offer a practical recommendation grounded in these considerations.

This paper is structured as follows. In Sect. 2, we provide a working definition of focus-sensitivity for clause-embedding predicates, which will serve as the theoretical basis for the discussion of different elicitation methods in the subsequent sections. In Sect. 3, we offer our practical recommendation for diagnosing focus-sensitivity. According to the recommended method, a consultant will first judge whether entailment holds between a pair of test sentences. In case the entailment is judged not to hold, a context that invalidates the entailment is elicited. This context is then used in a second part of the method, which utilizes a truth-value judgment. Within the same section, we provide an initial assessment of the method, in light of the general desiderata for semantic elicitation methods. In Sects. 4 through 7, we provide further assessment by comparing it to a number of alternatives: a method that uses a truth-based test alone, one that uses a coherence-based test, and one that uses an inference-based test alone. We end this comparison with a remaining issue for the two-step test that we propose. We conclude that these methods are less favorable than the recommended one with respect to one or more of our desiderata. Sect. 8 provides a general summary and outlook.

## The focus-sensitivity of clause-embedding predicates

Before discussing methods for diagnosing the focus-sensitivity of clause-embedding predicates, we provide in this section a working definition of focus-sensitivity for clause-embedding predicates. The definition is stated in (2).^2^

(2)
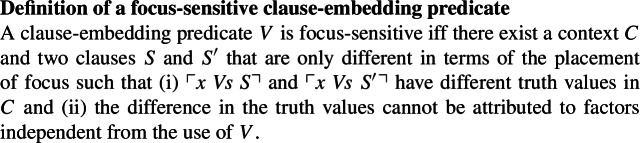

Part (i) in the definition straightforwardly captures the intuition that focus placement should have a truth-conditional effect for focus-sensitive predicates. Part (ii) ensures that it is indeed the clause-embedding predicate that is responsible for the sensitivity of the sentences’ truth values to focus placement. That is, we need to rule out other confounding factors that may introduce focus-sensitivity. For instance, if there is a focus-sensitive operator such as *only* and *even* in the embedded clause, Condition (i) will be met, but because of Condition (ii), we will not take such sentences as evidence for the embedding predicate being focus-sensitive.

One consequence of Condition (ii) is that, to establish the focus-sensitivity of a predicate, we should use *exhaustivity-securing* contexts, i.e., contexts that ensure that the wh-question congruent (in the sense of Rooth 1992) with the target sentence is answered strongly-exhaustively by the target sentence. To see why this is important, consider the following context (inspired by and modified from Villalta’s 2008 examples that are discussed in Sect. 4), together with sentences where the predicate *think* embeds clauses with different focus placements.

(3)
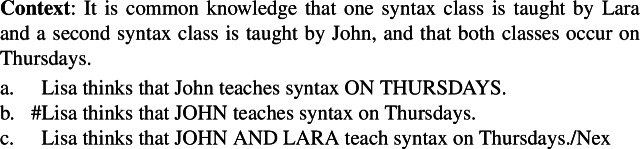

The reason behind the infelicity of (3b) is that focus on *John* strongly invites a reading under which the sentence is taken to be an exhaustive answer to the question “Which individual(s) are such that Lisa thinks that they teach syntax on Tuesdays?” and it is not the correct exhaustive answer in this context. In contrast, the sentence in (3c) with both instructors named and narrowly focused is felicitous (and true). This means that the contrast between (3a) and (3b) is due to the fact that there exist non-exhausitive answers such as *John teaches syntax on Tuesday* in the context, which is independent of the use of the predicate *think*. Therefore, in order to show a predicate is focus-sensitive, we need to consider an exhaustivity-securing context such as (4), where there are no non-exhaustive answers. Failing to do so could give rise to contrasts like the one between (3a) and (3b) that are indeed driven by the position of focus, but lead to the wrong conclusion that a predicate like *think* is focus-sensitive.

(4)
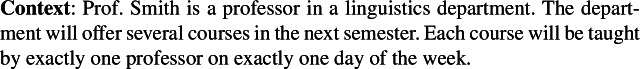

Unless stated otherwise, all of the contexts provided in this paper are exhaustivity-securing. Concretely, they always involve the background assumption that any given class has only one instructor, and that it occurs on only one day of the week.

## Diagnosing the focus-sensitivity of clause-embedding predicates: a field manual

In this section, we present a method for deciding whether an arbitrary clause-embedding predicate in an arbitrary language is focus-sensitive or not. It consists of a sequence of two tests, respectively called the “inference-based” and the “truth-based” tests, which can be described *in abstracto* as follows.

Take two sentences of the form (i) ⌜x Vs that [A …B_*F*_] ⌝ and (ii) ⌜x Vs that [A …] ⌝, where [A …B_*F*_] represents an embedded clause with a narrow focus on its syntactically optional sub-constituent B and [A …] is obtained by dropping B. The first step of the test is to directly ask if (i) entails (ii), and we expect this not to be an entailment if V is focus-sensitive (an assumption that we will discuss later in Sect. 7). If all consultants judge this inference to be an entailment, the procedure stops and the researcher concludes that there is no evidence that V is focus-sensitive (for any consultant).^3^ In contrast, if at least one consultant judges that this is *not* an entailment, a context *C* is elicited that makes (i) true and (ii) false (which we call an *entailment-invalidating* context or *invalidating* context for short), thus confirming lack of entailment, and the procedure moves on to a second test (for all consultants, including those who initially judged the inference to be an entailment). This second test involves a third sentence of the form (iii) ⌜x Vs that [A_*F*_ …B] ⌝, which is identical to (i) except for the placement of focus in the embedded clause. The researcher asks each consultant whether (i) and (iii) have the same true values in context *C*. If the consultant judges the truth values to be different, the researcher concludes that the predicate is focus-sensitive for this consultant. If the consultant judges the truth values to be the same, the researcher concludes lack of evidence for focus-sensitivity for this consultant. As will be discussed in Sect. 6, this second test can help filter out certain false positive and false negative results and improve the stability of the overall test.

Below we elaborate on the concrete formulation of these tests.

### Steps for diagnosing focus-sensitivity

#### Step 0: Prerequisites

We assume that the researcher has established how focus is realized in the language under investigation. We further assume that, before applying (the steps of) our focus-sensitivity test, the researcher has taken the necessary preparatory steps so that the consultants understand the notion of entailment, can distinguish an entailment from a highly plausible inference that is not an entailment, and, in the latter case, are able to provide a concrete invalidating context that blocks the inference (and we will provide one specific way of training the consultants in Sect. 6).^4^

#### Step 1: Inference-based elicitation of entailment-invalidating contexts

The first step in running the inference-based elicitation of entailment-invalidating contexts is to construct test sentences and formulate a broad context that they are uttered against. The context provided should be exhaustivity-securing, for reasons discussed in the previous section. Below is a concrete example (5).

(5) **Example of a broad context**
  Prof. Smith is a professor in a linguistics department. The department will offer several courses in the next semester. Each course will be taught by exactly one professor on exactly one day of the week. Lisa is a student in the department. She knows all the background information above, but she may or may not know the exact course schedule. That is, it is possible that she has full, no, or partial information about the course schedule.

Next, the consultants are presented with pairs of sentences used in the inference-based test for clause-embedding predicates, e.g., (6)for *believe* and (7) for *want*.^5^ Note that any clause embedding predicate can be used to construct such pairs, so long as they are coherent with the broad context in (5).

(6)

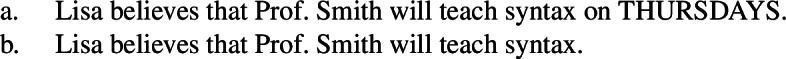

(7)

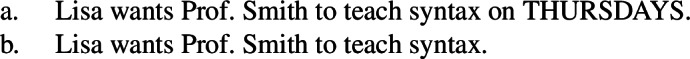

The consultants are then asked to judge whether the inference is an entailment, and if not, to provide an invalidating context, i.e., one that blocks the inference. For instance, in the case of *want* the consultants might provide the following invalidating context (8), where (7a) is true but (7b) is false.

(8) **Example of an invalidating context for** ***want***
  Lisa doesn’t really care about which professor will teach syntax, but she strongly prefers for the course to be taught on Thursdays. She doesn’t know yet when it will be, and she learns that Prof. Smith will teach it.

In contrast, in the case of *believe*, all consultants (presumably) will judge the inference (6) to be an entailment.

If all consultants judge the inference to be an entailment, the researcher will end up with an empty set of invalidating contexts. In this case our procedure stops here and the researcher concludes that there is no evidence for the predicate being focus-sensitive. If at least one consultant judges the inference to be a non-entailment and provides an invalidating context, the researcher proceeds to Step 2, which involves a truth-based test. Crucially, at Step 1, the consultants may provide different judgments. What is important is whether an invalidating context can be elicited at all, and Step 2 will be applied even to those consultants who judge the inference to be an entailment.

We note that, ultimately, the goal of Step 1 is to obtain a set of candidate contexts to be used in Step 2, and such contexts do not have to come solely from our inference-based test. In many cases, a researcher may well have good intuitions about the meaning of a predicate and the kinds of contexts that may show its focus-sensitivity, and they can certainly further include such contexts as candidates. Our point is that the inference-based test provides a principled way to elicit such contexts from consultants when the researcher does not have a good intuition about the focus-sensitivity status of a predicate in the target language. In Sect. 4, we present another potential way of generating contexts to be used in the truth-based test in Step 2, and show how it may be optionally incorporated into our workflow.

#### Step 2: Truth-based test

In this step, consultants are presented with the invalidating context(s) elicited in the previous step, e.g., (8) for *want*, repeated below in (9).

(9) Lisa doesn’t really care about which professor will teach syntax, but she strongly prefers the course to be taught on Thursdays. She doesn’t know yet when it will be, and she learns that Prof. Smith will teach it.

s They are then asked to judge whether the following sentences are true in this context.

(10)
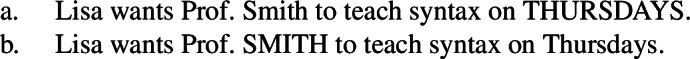

If these sentences differ in truth value (for at least one of the contexts elicited in Step 1), then the researcher concludes that the predicate is focus-sensitive. Otherwise the researcher concludes that there is no evidence for the predicate being focus-sensitive.

**Fig. 1:**
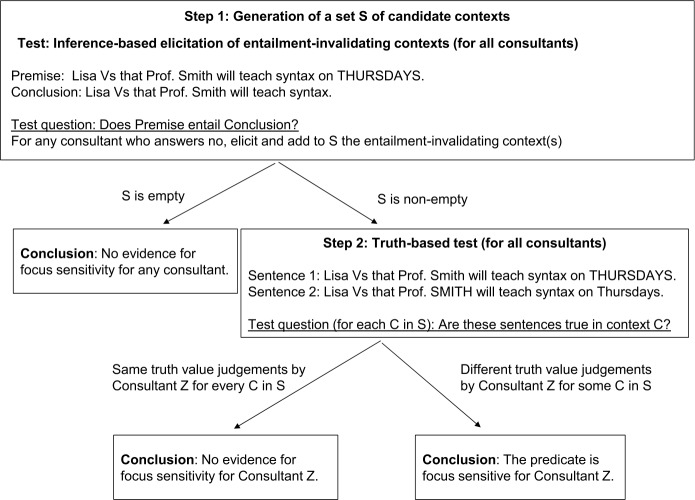
Workflow of the proposed two-step focus-sensitivity test

So far in this section, we have presented our proposed method for diagnosing focus-sensitivity of clause-embedding predicates, based on the combination of an inference-based diagnostics and a truth-based diagnostics, summarized in Fig. 1. In the rest of this section, following a general discussion of desiderata for methods of semantic data collection, we will provide an initial evaluation of the method.

### General desiderata for a test for focus-sensitivity

A methodology in semantic data collection can be evaluated in terms of specific desiderata. In this paper, we follow Tonhauser and Matthewson (2016) in adopting the following as criteria that semantic data collection methods should fulfill:

(11)
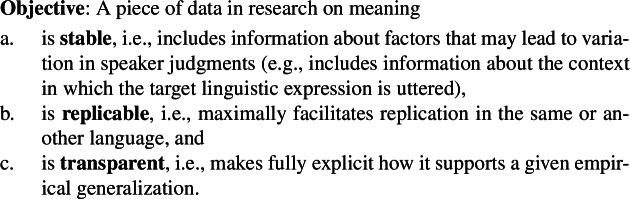

In addition to these general desiderata, we highlight the following more specific desiderata as additional considerations relevant for our evaluation of the methods of testing focus-sensitivity:

(12)
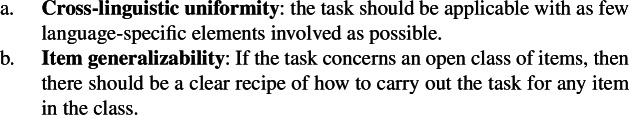

Two remarks are in order here. First, although the explicit formulations of the two desiderata in (12)are of our own, we do not claim that they are completely new or independent of the ones Tonhauser and Matthewson (2016) propose. In fact, arguably, they are entailed by **replicability** (11b). We highlight the desiderata in (12)as they are among the most relevant ones for our current purposes, i.e., cross-linguistic comparison. This said, we also emphasize that explicitly discussing these desiderata in the context of cross-linguistic semantic data collection is part of our original contribution.

Second, the properties relevant for these desiderata should be understood as graded. It may be impossible to have a task that is perfectly uniform or generalizable, and so language- or item-specific adjustments may be required in some cases. Positing (12)as desiderata, however, commits us to prefering a method that keeps such language-/item-specific adjustments as low as possible, so that we achieve as much **cross-linguistic uniformity** and **item generalizability** as practically possible. Similarly, regarding **stability**, Tonhauser and Matthewson’s discussion primarily emphasizes the necessity of including information about the context in which the critical sentence is evaluated and information about the native speaker making the judgment, but there may well be a number of additional factors that can lead to variation in speaker judgments. Therefore, a method may not be fully stable even if it provides information about the speaker and the context. Nevertheless, all else being equal, we should prefer methods that provide such information to those that do not. For the purpose of evaluation and comparison with various alternative approaches, the relevant factor we will consider in this paper regarding **stability** is whether a test produces data that have information about the context that may lead to variation in speaker judgments.

### Evaluation

Based on the desiderata outlined above, we now evaluate the diagnostic we presented in Sect. 3.1. In Sects. 4 through 7, we will further evaluate it by comparing it with a number of alternative approaches.

Due to the combination of eliciting invalidating contexts using an inference-based test and the subsequent truth-based test, this test is **stable**, in the sense of satisfying the necessary condition of providing relevant contextual information. (This will be further discussed when we compare it with some alternative approaches in later sections.)

This test is replicable. In particular, it satisfies both of our specific criteria: **cross-linguistic uniformity** and **item generalizability**. First, the test is cross-linguistically uniform, since it only involves notions of entailment and truth, which we take to be universal. Indeed, it is possible that a target language might lack *specific terms* for these concepts, but crucially the formulation of the test does not require the existence of such terms in the target language. What is required is the ability for the researcher and the consultant to refer to these concepts in the contact language through an appropriate preparatory step (see Step 0 in Sect. 3.1), which we take to be possible.^6^ Second, it guarantees **item generalizability**, due to the fact that there is a general recipe that can be applied to any predicate.

This test is mostly **transparent**, in the sense that it is designed to diagnose focus-sensitivity as defined in Sect. 2. In particular, when the researcher concludes that a predicate is focus-sensitive (for a consultant), they have the sentences and the context required by the definition to support this conclusion. That is, this test never yields false positives. However, in Sect. 7 we will discuss cases of potential false negatives, i.e., focus-sensitive predicates that fail to be categorized as such.

As we mentioned, the properties relevant for these desiderata are graded. Thus, whether a certain diagnostic meets them to a satisfactory degree should be considered in comparison with alternatives. In the next section, we will consider alternatives to the proposed diagnostic, including using the inference-based test alone and using the truth-based test alone. As it turns out, each one of these alternatives fails to satisfy some of the desiderata listed above. Furthermore, as we will see, the issue regarding the potential false negatives is a problem also for an approach that only utilizes the truth-based test or the inference-based test, making the approaches at least as non-transparent as the proposed test.

In this section, we have presented a method for diagnosing the focus-sensitivity of clause-embedding predicates that is designed to meet the general desiderata for collecting semantic data outlined in Sect. 3.2. Crucially, the method combines a *truth-based* test that is traditionally used in the literature on focus-sensitivity (Villalta 2008) and an *inference-based* elicitation of context. At this point, it is natural to question why this relatively complex methodology should be adopted, and whether it is sufficient to employ a simpler method consisting of only the truth-based test or only the inference-based test. In the following sections, we address these questions by considering three potential alternatives: using the truth-based test alone (Sect. 4), using a coherence-based test discussed by Villalta (2008) instead (Sect. 5), and using the inference-based test alone (Sect. 6). We will argue that these alternatives fail to meet one or more of the desiderata listed in Sect. 3.2, and thus are less preferable than our proposed method as a general method for detecting focus-sensitivity. This, however, does not mean that our proposed method is problem-free. In Sect. 7, we discuss remaining issues with our two-step methodology.

## A challenge for using the truth-based test alone: item generalizability

Testing for the focus-sensitivity of an arbitrary predicate *V* using the truth-based test uses the following procedure:

(13)
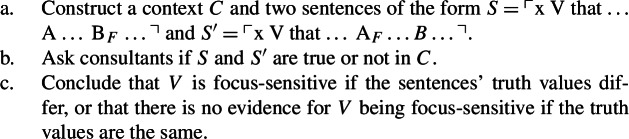

Whether a truth-based test can be run following this procedure for any choice of *V*—in other words, whether it is item generalizable—depends on whether a general recipe *R* can be formulated for constructing *C*. This is the question that we address in this section.

We entertain an initial candidate for *R* based on Villalta’s (2008) original contexts but set it aside mainly on the basis of the fact that it does not generalize to non-preferential predicates (regardless of whether they might be focus-sensitive or not). We then suggest an alternative recipe that *is* able to generate a context for any *V* in a non-arbitrary way, but show that the contexts generated in this way cannot be used to elicit truth-value judgments directly: the researcher using this recipe must first establish that the context is not self-contradictory. This, in turn, leads to a test for focus-sensitivity that is not based on truth-value judgments alone, but to a two-step variant, comparable to the method outlined in the previous section.

### Original formulation of the truth-based test

The truth-based test presented in Sect. 3.1 is originally found in Villalta (2008: Sect. 7.1), then later in Romero (2015) and Uegaki and Sudo (2019). These authors mostly use this test alone, though Villalta does briefly consider an additional coherence based test, which we discuss in Sect. 5.

Here is Villalta’s original context and target sentences for the predicate *want* (Villalta 2008: Sect. 7.1). We divide it up into a “broad” component, which specifies the target sentences’ relevant focus alternatives, the attitude holder’s beliefs and other facts, and an “immediate” component, which specifies the relevant preferences that the attitude holder bears to parts of the embedded proposition. (We will later simplify these contexts and target sentences.)

(14)
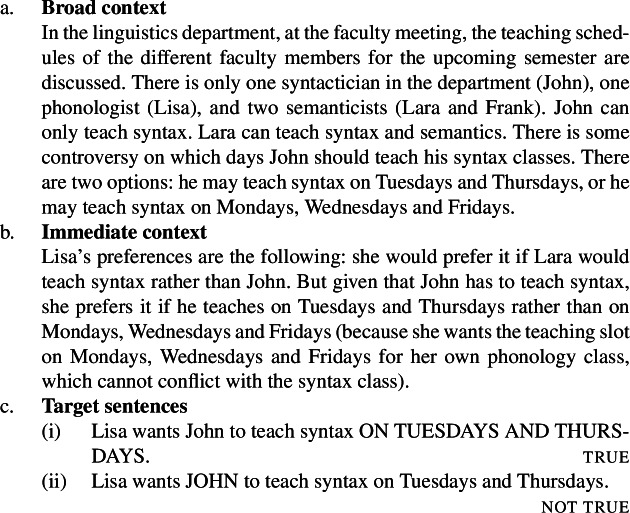

In this context, (14c-i) is judged true, while (14c-ii) is judged not true.^7^ This suggests that the position of focus under the scope of *want* gives rise to truth conditional differences, which motivates the conclusion that *want* is a focus-sensitive predicate.^8^

Villalta then considers the emotive factive predicate *be glad* and modifies the (broad) context so that the predicate’s factive presupposition is satisfied. Instead of “[t]here is some controversy on which days John should teach his syntax classes” as in (14a), we assume, citing Villalta, that “at the end of the faculty meeting, *it is decided that* John is indeed going to teach syntax on Tuesdays and Thursdays”.

(15)
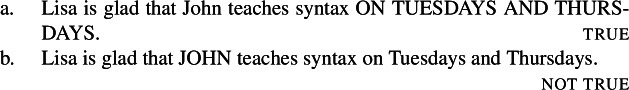

Given Lisa’s preferences as specified in (14b), here too, (15a) is judged true and (15b) not true, motivating the conclusion that *be glad* is a focus-sensitive predicate.

Finally, Villalta contrasts the situation with *want* and *be glad* with what happens with the factive doxastic predicate *know*. About the pair of sentences in (16), she writes that “[they] are both true under the same circumstances: all contexts that make one of them true make the other true as well”, which amounts to saying that they are truth conditionally equivalent.

(16)
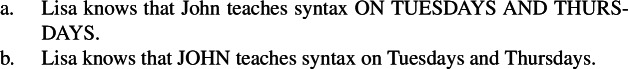

Because the position of focus under the scope of *know* does not give rise to a truth conditional difference, the predicate is classified as non-focus-sensitive. As additional evidence for the non-focus-sensitivity of *know*, Villalta presents a coherence based test as well, which we discuss in Sect. 5.

While an appendix in Villalta (2008) contains a long and varied list of predicates coded for whether they are focus-sensitive or not, no general method is given for constructing the contexts to be used in a truth-based test for focus-sensitivity. But contexts are necessary for a consultant to be able to give truth-value judgments (Matthewson 2004) and we have seen (in moving from *want* to *be glad*) that these will have to vary from predicate to predicate. We would thus like to know if it is possible to formulate a recipe for constructing contexts for any choice of attitude verb.

### It is not always about conflicting preferences on a substrate of belief

Take the immediate context and the judgments for the pair of *want* reports in (14). The reason that the context makes the first target sentence (14c-i) true, and the second one (14c-ii) false is that it sets up a conflict between one desire (for Tuesdays) and another (against John). This property of Villalta-style contexts is stated in (17)as *the conflicting attitude requirement* (on the immediate context). While the original context gives the attitude holder a negative preference, just a lack of preference (or indifference) is sufficient to make the second sentence false.

(17)
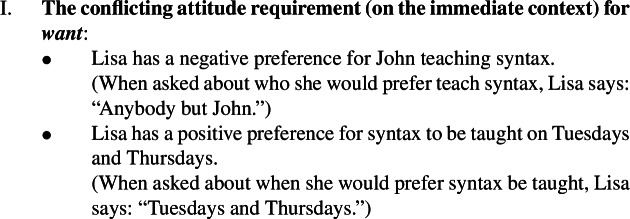

In the case of *want* and *be glad*, and preferentials in general, the conflicting attitude requirement seems to reference the main semantic component of the attitude verb: the agent’s preferences.

The same context also imposes a requirement on Lisa’s beliefs, namely, the requirement that she believe that John will be the teacher. This second property of Villalta-style contexts, we state in (18)and call *the substrate attitude requirement* (on the immediate context).

(18)
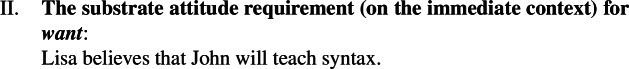

These two requirements need to be filled in by statements that are consistent with each other. In the case of preferentials, consistency is possible because the conflicting attitude and the substrate requirements involve different modalities, respectively bouletic and doxastic, and having a negative preference for John to be the teacher does not contradict the belief that he will be.

Now, the substrate attitude requirement is necessary for the test to work with *want*, where “to work” means that we want the predicate to come out as focus-sensitive. To see this, consider the possibility that it has *not* been decided who will teach syntax. This now implies that Lisa has no particular beliefs about who will teach syntax. But, assume that she has a dispreference for John and a preference for the Thursday slot. (From now on, we simplify the context and the target sentences so that they only talk about Thursdays.)

(19)
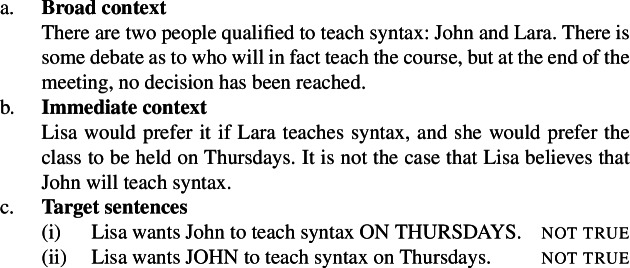

In this context, neither sentence in (19c) is true. In particular, the intuition for (19c-i) is that the mention of *John* in the embedded clause is enough to attribute to Lisa the belief that he will teach syntax, and this conflicts with the broad context: Lisa knows that it hasn’t been decided who will teach syntax. Without the substrate attitude, then, we cannot make the first sentence true and we cannot test whether shifting focus elsewhere in the embedded clause affects truth conditions. To repair (19c-i), one could use a definite description (“Lisa wants the person who will teach syntax to teach it on Tuesdays”) or a passive construction (“Lisa wants syntax to be taught on Tuesdays”). But then we cannot construct a minimally different sentence with focus on the subject.

Parallel sentences with *be glad* require that Lisa believes that John will teach syntax on Tuesdays, where *on Tuesdays* is added to the belief. This requirement stems at least from emotive factives’ belief presupposition: *x is glad that p* presupposes that x believes p (Klein 1975; Egré 2008, a.o.). And in contexts in which this presupposition is not satisfied, *x is glad that p* will generally be infelicitous. In this case, the substrate attitude coincides with or is entailed by this presupposition.

Given what we know about these predicates’ presuppositions and other semantic properties, it is likely not a coincidence that the substrate attitude corresponding to *want* and *be glad* assumes a background belief, while setting up a conflict between the subject’s preferences: preferential predicates are traditionally taken to induce a preference-based ordering on a modal base consisting of an agent’s doxastic alternatives (Heim 1992; von Fintel 1999). The ingredients for constructing Villalta-style contexts for these predicates are the same ones, but served differently.

At this stage, one might think that a general recipe for constructing Villalta-style contexts always involves determining a belief for the substrate and a pair of conflicting preferences. But this cannot be the case. The predicate *be surprised*, for example, is listed as focus-sensitive by Villalta and the attempt to construct a context for it reveals that it needs a belief substrate but a pair of conflicting *likelihood judgments* rather than preferences. This is shown by the context and pair of sentences in (20).

(20)
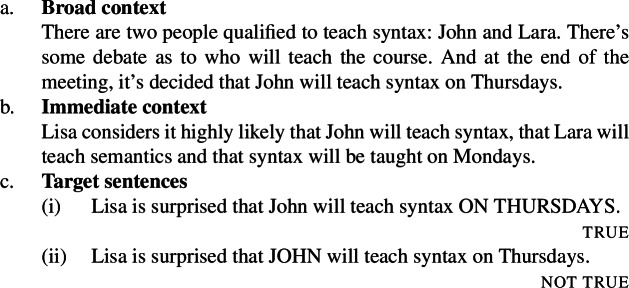

Note that if the conflicting attitudes did not specify likelihood but instead were preferential, the test would not have worked in that the context would not have enough information to make the sentences true or false.

There are also attitude predicates where the conflicting and the substrate attitudes look very different. These mostly concern speech predicates like *guess*, *answer* or *repeat*. The following example is modified from Romero (2013), who argues for the focus-sensitivity of *guess* using a Villalta-style truth-based test.

(21)
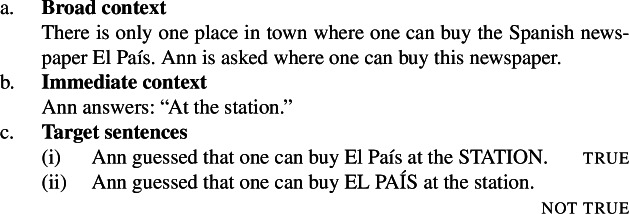

We do not know what the range of possible substrate and conflicting attitudes are, but these two examples suffice to show that the truth-based test does not always involve a belief substrate with a pair of conflicting preferences. Hence, one can’t naïvely transpose elements from the *want* and *be glad* contexts onto ones for other predicates, even if they might be focus-sensitive. In the absence of a general recipe, the truth-based test cannot be run in an item-generalizable way.

### A non-arbitrary way of determining the substrate and conflicting attitudes?

In the case of *be glad*, above, we pointed out that the substrate attitude also corresponds to the predicate’s belief presupposition, namely to the belief that the prejacent of *be glad* is true. Additionally, in both the *want* and the *be glad* cases, the conflicting attitudes were preferential, corresponding to the fact that these predicates assert something about an agent’s preferences.

There might be a general pattern here, which has the potential of yielding a general recipe for constructing truth-based tests for focus-sensitivity. (We thank one of our reviewers for bringing up this possibility and for sketching out the discussion in this section.) The recipe goes like this:

(22)
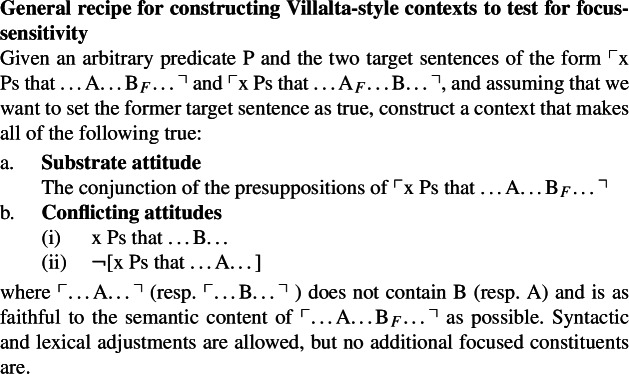

Ex. (23)illustrates with the predicate P = ‘be surprised’, for which a test context is not provided by Villalta (2008), to suggest that the recipe seems to generalize. Setting A = ‘John’ and B = ‘on Thursdays’, we get:

(23)
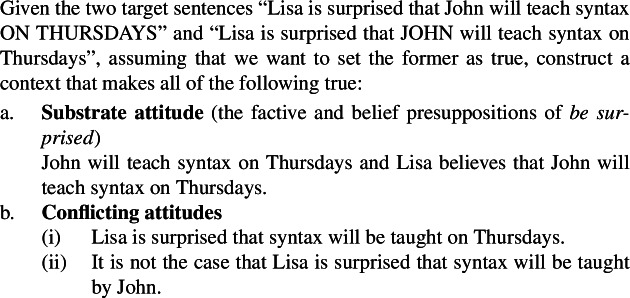

In this context, the judgments for the target sentences given are as follows: (24a) is true and (24b) is not, which suffices to classify *be surprised* as a focus-sensitive predicate. A welcome result.

(24)

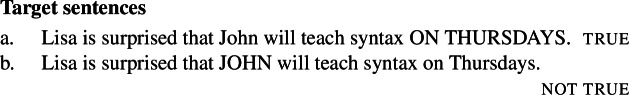

The applicability of this method for determining suitable substrate attitudes crucially depends on the researcher’s knowledge of the relevant presuppositions of sentences of the form ⌜x Vs that S⌝, which might not always be straightforward. We illustrate this point with *want*. Recall from the discussion of this predicate, in (17) and (18), that its substrate attitude is belief. In other words, ⌜x wants … A … B_*F*_ …⌝ should be judged in a context in which x believes that A. For the general context construction method to predict this, it has to be the case that the desire report presupposes the belief report. This is perhaps expected given two independent properties of focus and desire reports. First, it is sometimes argued that (25a) presupposes (25b), i.e., that focus triggers an existential presupposition (Geurts and van der Sandt 2004).

(25)
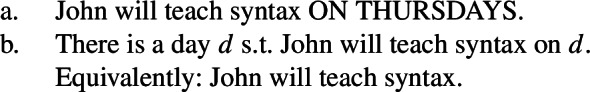

Second, Heim (1992), attributing the observation to Karttunen (1973, 1974), argues that attitude reports of the form ⌜x Vs that p⌝, where V is a doxastic or a preferential predicate, presuppose that x believes the presuppositions of p. In the case of *want*, for example, (26a) is taken to presuppose (26b).

(26)
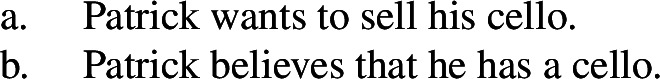

If, then, the embedded sentence in (27a) comes with the existential presupposition in (25b), we expect it to presuppose (27b) and the general method for constructing contexts is successful in predicting an appropriate substrate attitude.

(27)

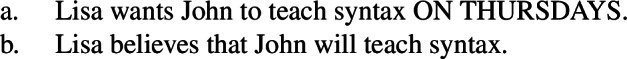

Setting aside challenges related to calculating the substrate attitude based on the presuppositions of a predicate, a separate issue arises when we extend this method to a predicate like *believe*. Indeed, both of the observations illustrated with *want*, namely the focus existence presupposition and accommodating a sentential complement’s presuppositions in the attitude holder’s belief state, carry over to this predicate too. The method for constructing contexts then says to construct a context that makes all of the following true.

(28)
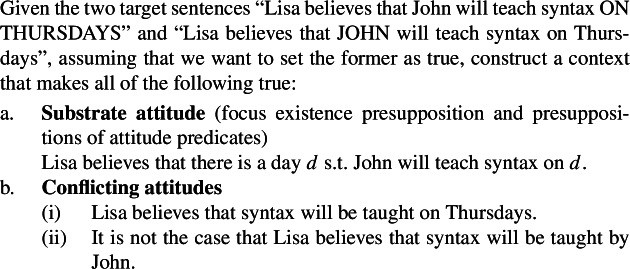

Here, the second conflicting attitude contradicts the substrate attitude that is obtained by considering the presuppositions of the true target sentence. This then makes it impossible to construct a usable Villalta-style context for the predicate *believe*: contexts in which consultants are asked to provide truth-value judgments should not contain contradictory information. In light of this issue, our reviewer suggests the following procedure (29)for how to run a truth-based test based on the general recipe in (22):

(29)
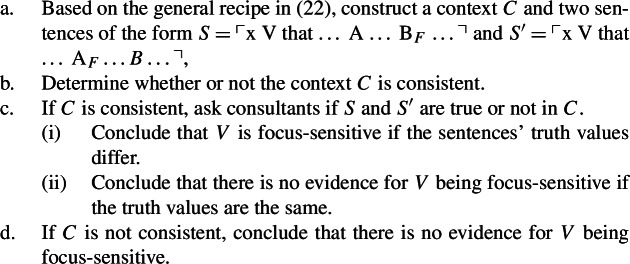

We note, however, that whether or not the context *C* is consistent can only be determined by a consultant, and not (always) by the researcher themselves because the conflicting attitudes (22b) are stated in the target language, which the researcher doesn’t necessarily have intuitions about. This procedure then requires that the researcher run an additional consistency check with their consultant before being able to run a truth-value judgment task.

### An assessment of the general method for constructing contexts

We started out this subsection by asking whether a *truth-based test only* method could be used to test for focus-sensitivity in an item general way. The answer is negative (as far as we can tell).

First, the procedure just outlined is only item general *to the extent that* the researcher is able to determine the relevant presuppositions of the predicates that they are testing in a general way, as this is required for the recipe in (22) to work. It remains unclear to us, however, whether this is generally an easy task: consider that even the presuppositions of very familiar predicates like English *know* are under active discussion. Compare its textbook treatment, where it is expected to presuppose its complement, to, e.g., Hazlett (2010), where it doesn’t, to, e.g., Degen and Tonhauser (2022), from which one can conclude, for the topic at hand, that presupposition is a difficult notion to work with.

Second, the procedure does not constitute a truth-based *only* test. It also requires that the researcher first ask the consultant whether the context that they have constructed by means of the general recipe is consistent or not. Only if the context is judged to be consistent can it be used to run a truth-value-judgment task proper. This means that the procedure for running the test in (29), even if it is based on a single general recipe for constructing contexts, is a two-step procedure. And considering the fact that checking whether any *p* and *q* is consistent is equivalent to checking whether *p* entails ¬*q*, the resulting test here is not so different from the two-step proposal we proposed in the previous section.

But, granted the recommendation that a pair of truth-value judgments should always be elicited in the process of determining whether or not a predicate is focus-sensitive—see, e.g., Sect. 6 for why we can’t rely solely on an inference-based test for this purpose—the inference and the general recipe-based tests both serve the same purpose of generating contexts intended to be used for this task. Two tests are better than one, and these are not so different in their logical underpinnings and in the steps that they might require from consultants: evaluating a pair of sentences and judging a certain entailment relation between them, or evaluating a context, and judging its consistency.

**Fig. 2:**
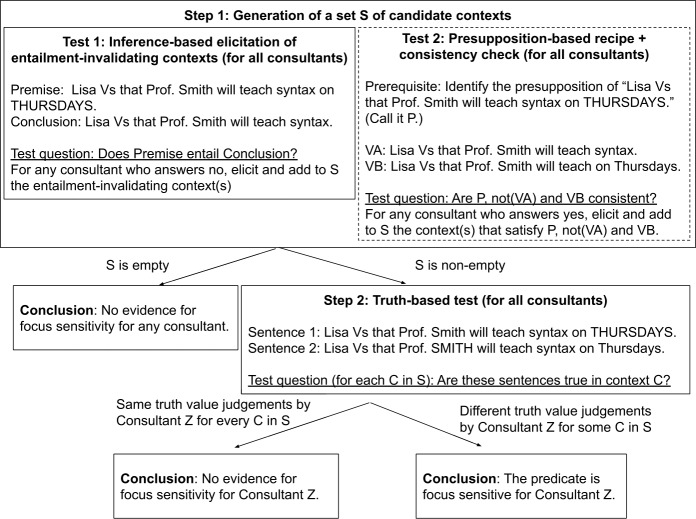
A possible refinement of the workflow of the proposed focus-sensitivity test with an optional Test 2 in Step 1 that may increase the chance of generating relevant candidate contexts

We thus suggest that Step 1 of our proposal can (and sometimes should) take on many forms, one of which makes use of the general recipe based method in (29)in addition to the inference-based test. The flowchart that summarizes this possible refinement to the method presented in Sect. 3.1 is in Fig. 2.

## Problems with using a coherence-based test

The second alternative test for focus-sensitivity involves judging whether a piece of dialogue is coherent or contradictory. We will refer to this test as the *coherence-based test*.

Villalta (2008, pp. 497–498) introduces what we will call a *minimalistic* variant of the coherence-based test. Her examples illustrate a contrast between *know* and *want*. For *know*, she points out that B’s reply to A in (30)is contradictory.

(30)
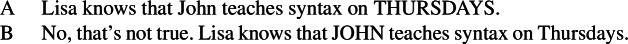

This is in contrast with *want*, for which B’s reply in (31)is judged to not be contradictory.

(31)
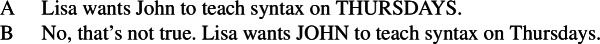

In general, if a predicate *V* is focus-sensitive, given the definition (2), there exist a context *C* and two embedded clauses *S* and $S'$ that only differ in the placement of focus such that ⌜*x Vs S*⌝ and ⌜*x Vs* $S'$⌝ have different truth values in *C*. Therefore, it should be coherent for one to deny the former and assert the latter instead.

However, not all speakers find (31)coherent. In other words, the minimalistic variant of the coherence-based test fails to meet the **stability** desideratum (11). In fact, Villalta (2008, p. 498, fn. 11) acknowledges herself that “[f]or some speakers, a more explicit context is necessary to make this a natural dialogue”. This motivates her to further introduce another variant of the coherence-based test, which she attributes to a personal communication by Jenny Doetjes. We will call it the *naturalistic* variant. In this variant, B’s provides more elaboration in their reply, which we present in bold in (32).

(32) B: Well, that’s not really true, **as she doesn’t mind which day these classes take place, as long as John is the one who does the teaching**, so one should rather say that Lisa wants JOHN to teach syntax on Thursdays.

While this additional contextual information makes the dialogue more natural and more clearly consistent, we note that it is predicate specific. Consider *decide* for instance. What would be an appropriate context to include in the dialogue? A simple-minded substitution based on (32)would be nonsensical (33).

(33)
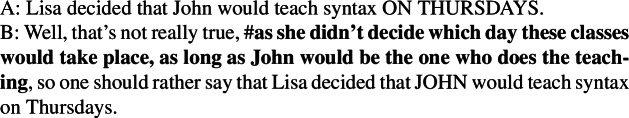

Of course, we should not conclude from the infelicity of (33)that *decide* is not focus-sensitive, because we can make the dialogue coherent by using a different context (34).

(34) B: Well, that’s not really true, **as she didn’t decide which day these classes would take place—that part had been determined long ago by someone else**, so one should rather say that Lisa decided that JOHN would teach syntax on Thursdays.

Note, however, that the second half of the context needs to be adjusted in a predicate-specific way. Therefore, the naturalistic variant of the coherence-based test faces the same limitation as the truth-based test discussed above in that there is no general recipe to construct the relevant contexts. That is, it falls short on **item generalizability** (12b).^9^

Moreover, in our experience, even the “naturalistic” variant is not so natural after all. Even for canonical focus-sensitive predicates such as *hope* and *want*, for which we can directly use relevant contexts in the literature, our consultants often found the dialogues complicated and artificial. Of course, it may well be that we were facing this difficulty because we were not using the most natural dialogues. But this in fact illustrates another limitation of the test, i.e., it is difficult to know what dialogues would be good to use. What counts as a good dialogue will probably differ from language to language. In addition, because the naturalistic variant requires an explicit context in the dialogue, such a context needs to be properly translated into the target language and integrated into the dialogue. This will likely involve language-specific adjustments that will reduce the uniformity of the test across languages. Due to the predicate-specific and language-specific adjustments required for the test, the test does not completely satisfy the **item generalizability** and **cross-linguistic uniformity** desiderata.

Finally, both variants of the coherence-based test involve a denial in the dialogue. This raises a general concern about what the denial is doing and whether it is doing the same thing across languages. This is particularly so for the minimalistic variant in (30). The consultant might judge the dialogue consistent by imagining a context where Lisa has a preference for John to teach syntax as well as a preference for it to be on Thursdays, but the Question Under Discussion (QUD, à la Roberts 2012) is about Lisa’s preference about who will teach syntax on Thursdays rather than her preference about when John will teach syntax. In such a context, B’s response can be seen as a metalinguistic move to correct A’s placement of narrow focus just to ensure that it is congruent with the QUD, and therefore it does not provide evidence that the predicate *want* is focus-sensitive. While this possibility might not be available in the English version of (30) because B’s response uses “that’s not true”, there is no guarantee that the target language has such a truth predicate, or that it is natural to use it in a denial. For instance, if in the target language it is only possible to use a counterpart of “that’s incorrect” in English, then B’s response can be more easily judged consistent under the metalinguistic correction interpretation above. As a result, all predicates may appear to be focus-sensitive due to the general constraint on focus-question congruence, which is not the kind of focus-sensitivity we are interested in (recall our definition in (2)). Of course, one can always ask consultants to rule out such metalinguistic corrections when judging the consistency of the resulting dialogue. However, the distinction can be quite subtle, and is likely to make the results less comparable cross-linguistically. For the naturalistic variant (34), the concern that B’s correction is about a general felicity condition on focus-QUD congruence is alleviated because the additional context makes it clearer that the correction rather has to do with the meaning of the specific predicate *decide*. But note that B’s response uses explicitly metalinguistic comments: *well*, *not really* and *one would rather say*. While such expressions can help make the dialogue more natural, they may well introduce their own idiosyncratic effects that reduce the transparency and cross-linguistic uniformity of the test. All in all, the involvement of denial in the test makes it more difficult to achieve **transparency** and **cross-linguistic uniformity**. A test that avoids such confounding factors altogether would be better.

In sum, the coherence-based test probes for focus-sensitivity by checking whether it is consistent to deny a sentence with narrow focus in one position and assert a string identical sentence with narrow focus in a different position.^10^ However, it is difficult to construct good dialogues (let alone creating a general recipe for constructing good dialogues for any given predicate in any given language) that sound natural and guarantee that the denial is not just a metalinguistic correction. This is particularly so for the minimalistic variant. In order to make the dialogues more natural, researchers need to provide more elaborate contexts, but again there is no general recipe to construct such contexts. Overall, we conclude that the test may fail the **stability** (for the minimalistic variant), **item generalizability** (for the naturalistic variant), as well as **transparency** and **cross-linguistic uniformity** (for both variants) desiderata and thus is less suitable as a general method than our proposed method.

## Problems with only using an inference-based test

A third possibility of testing focus-sensitivity using only a single task is to run an inference-based test alone without a follow-up step that utilizes the truth-based test. We consider two variants of such a test.

The *1-inference* variant determines whether a predicate is focus-sensitive solely based on the consultant’s judgment about the inference used in the first step of our proposed test. That is, a consultant would be introduced to a broad context such as the following (35), repeated from (5).

(35)
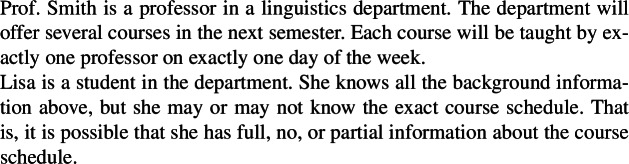

Against this broad context, the consultant is asked to judge whether (36a) entails (36b).

(36)
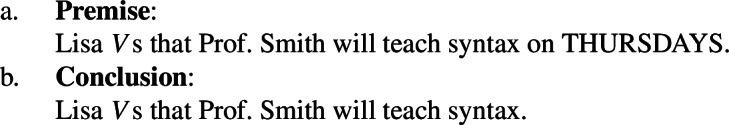

If the consultant judges this inference to be an entailment, the researcher concludes that there is no evidence for this predicate being focus-sensitive for this consultant. In contrast, if the consultant judges this inference not to be an entailment, the researcher concludes that the predicate is focus-sensitive for this consultant.

The second variant, which we call the *2-inference* variant, involves an additional judgment about the following inference, whose premise (37a) is different from the previous one (36a) only in the placement of focus in the embedded clause and whose conclusion (37b) is the same as before (36b).

(37)
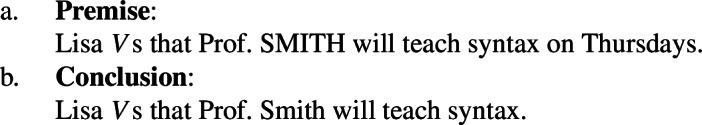

If the consultant’s judgments about the two inferences are different (i.e., one is judged to be an entailment and the other is not), the researcher concludes that the predicate is focus-sensitive for this consultant. In contrast, the consultant’s judgments about the two inferences are the same, the researcher concludes that there is no evidence for the predicate being focus-sensitive for this consultant.

The idea behind the 2-inference variant is the following. If the two premises, (36a) and (37a), which only differ in the placement of focus in the embedded clause, have the same truth conditions, then they should have identical inference patterns. That is, for any sentence, either they both entail it or neither does. Now, suppose that, contrary to this expectation, the researcher discovers that only one of the premises entails (36b)/(37b). Then they can take this as evidence that the two premises in fact do not have the same truth conditions and conclude that the predicate is focus-sensitive.

Clearly, both variants are **cross-linguistically uniform** and **item generalizable**, at least as much so as our proposed test. However, as we discuss below, they fall short on **transparency** and **stability**.

### Non-upward-entailing predicates: potential false positives/negatives

The first limitation of only using the inference-based test is that it may lead to incorrect conclusions for non-upward-entailing (non-UE) predicates. The two variants are prone to different types of errors and we discuss each of them below.

First, for the 1-inference variant, consider the following predicate $D'$. Its meaning, defined in (38), loosely resembles that of the English predicate *deny*, but we are not committed to this being a fully adequate analysis of *deny*. For our current purposes, what matters is that the meaning of $D'$ seems reasonable and therefore it is conceivable that $D'$ may be lexicalized in some natural language.

(38)
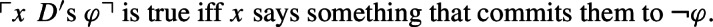

Given that the meaning of $D'$ as defined in (38)has nothing to do with the focus placement in *φ*, $D'$ is not focus-sensitive.

Now consider the following scenario (39).

(39)
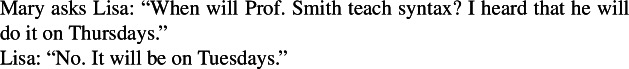

In this scenario, based on the meaning of $D'$, we can verify that (40a) is TRUE and (40b) is not. Therefore, when asked whether (40a) entails (40b), barring performance errors, a consultant would judge the inference not to be an entailment. Consequently, the 1-inference variant would classify $D'$ as focus-sensitive, contrary to fact. In other words, the 1-inference variant yields a false positive for $D'$.

(40)

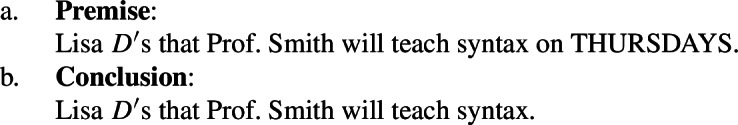

In comparison, if the researcher instead applies our proposed test, they would elicit the invalidating context (39)from the consultant and proceed to the second step, where the consulant judges the truth values of (40a) and (41)in this context.

(41) Lisa $D'$s that Prof. SMITH will teach syntax on Thursdays.

Given the meaning of $D'$ (38), both sentences would be judged true, and therefore the researcher would (correctly) conclude that there is no evidence for $D'$ being focus-sensitive. In other words, our proposed test can avoid a false positive for $D'$, thanks to the second step that uses the truth-based test.

The 2-inference variant would correctly conclude that there is no evidence for $D'$ being focus-sensitive, but it suffers from a different type of error, which we illustrate below. Consider a slightly different predicate $D''$, which has a focus-sensitive meaning defined as follows (42).

(42)
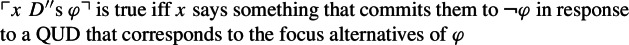

We first observe that, in the same scenario above (39), (43a) is true but (43b) is not. Therefore, (43)is not an entailment.

(43)
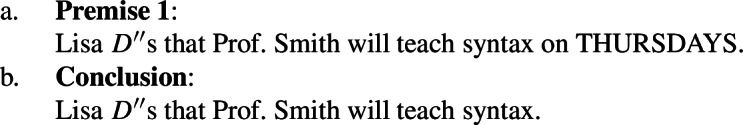

Now consider a different scenario (44).

(44)
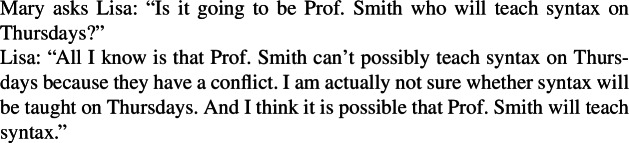

In this scenario, (45a) is true and (45b) is not.^11^ Therefore, (45)is not an entailment, either.

(45)
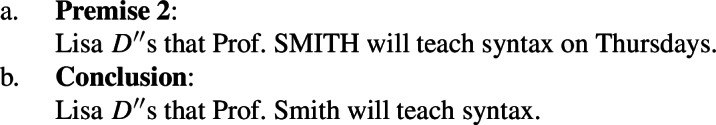

Given that the judgments about the two inferences are the same (i.e., neither is an entailment), the 2-inference variant would conclude that there is no evidence for $D''$ being focus-sensitive. But in reality, $D''$ is focus-sensitive, because in scenario (39), Premise 1 (43a) is true but Premise 2 (45a) is not. Therefore, the 2-inference variant yields a false negative for $D''$.^12^

In comparison, if the researcher instead applies our proposed test, they would elicit the invalidating context (39) and proceed with a truth-based test, where the consultant judges the truth values of (43a) and (45a) in this context. Since the former is true but the latter is not, the researcher would (correctly) conclude that $D''$ is focus-sensitive. Therefore, our proposed test can avoid a false negative for $D''$, thanks to the second step that uses the truth-based test.

In sum, if the researcher only applies the inference-based test, regardless of which variant they use, the test may yield incorrect results for non-UE predicates such as $D'$ and $D''$: the 1-inference variant yields a false positive for $D'$ and the 2-inference variant yields a false negative for $D''$. In comparison, such errors can be avoided by our proposed test due to the additional step of applying the truth-based test. It is in principle possible that problematic predicates such as $D'$ and $D''$ are in reality unattested in natural languages, making it safe to only apply the inference-based test. However, this possibility does not seem very plausible to us. And even if it were true, it is unclear why it should hold a priori. That is, only applying the inference-based test is less **transparent** than our proposal. Therefore we conclude that our two-step approach should be preferred.

### Difficulty in detecting (certain) non-entailments

The second limitation of only applying the inference-based test concerns its practical feasibility and stability. The test crucially relies on the consultants’ ability to determine whether an inference is an entailment (under certain contextual assumptions), i.e., whether the premise (contextually) entails the conclusion. Furthermore, when an inference is not an entailment, consultants need to be able to construct and articulate relevant invalidating contexts.

These can make the inference-based test more demanding for the consultants than the truth- and coherence-based tests. This is one of the reasons for why tests based on judgments about entailment are quite unusual in fieldwork, and why Tonhauser and Matthewson (2016) rate such tests towards the lower end of a scale based on linguistic tests’ stability, replicability and transparency.

#### Case Study

In light of this general concern about the practical feasibility of the inference-based test, We conducted an initial investigation with three non-linguist native speakers of varieties of British English.^13^ Each consultant, at the beginning of their respective elicitation session, was told that they would see pairs of sentences and that their task was to determine whether the second sentence necessarily follows from (the truth of) the first.

To familiarize them with the basic structure of the task, the consultants were first presented with Chierchia and McConnell-Ginet’s (1990) textbook examples of entailment (48) and (49), as well as highly plausible inferences that are not entailments (46) and (47). For each pair of sentences, the consultants were asked the following question: “Suppose the first sentence is true. Does it necessarily follow that the second sentence is also true?” All three consultants correctly answered NO for (46) and (47) and YES for (48) and (49). They were also asked to provide explanations for their judgments of non-entailment, i.e., for NO judgments, along the lines of “It could be sunny and cold”.

(46)

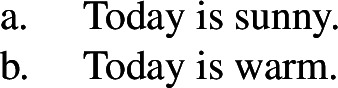

(47)

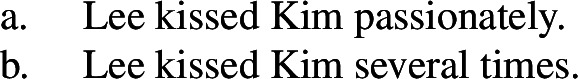

(48)

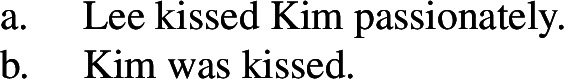

(49)

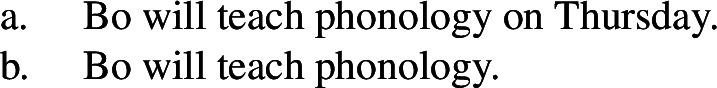

Next, the consultants were presented with the inference-based test for the additive particle *too*. They were asked to read out the first sentence in the pair with stress or emphasis on the word in capital letters and then were asked whether the truth of the second sentence necessarily follows from the first one.

(50)

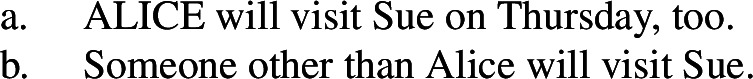

(51)
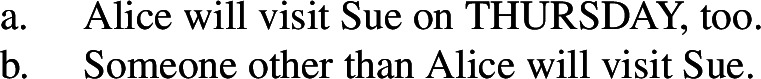

While Consultants 1 and 3 correctly answered NO for (51), Consultant 2 initially judged (51) to be an entailment. However, when we followed up by asking whether (51a) is compatible with the sentence *nobody else will visit Sue*, which is equivalent to the negation of (51b), Consultant 2 correctly answered YES and agreed that (51b) in fact does not necessarily follow from (51a).

Having finished the two practice questions with *too* (and the follow-up discussion, if there was any), the consultants were presented with the following broad context (52), repeated from (5), and were told that this would be the general background throughout the remainder of the consultation session.

(52)
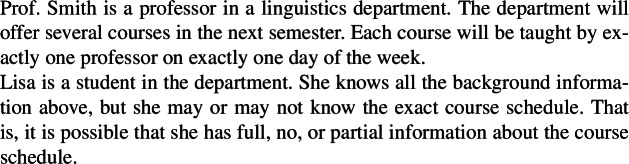

Finally, the consultants were presented with pairs of sentences used in the inference-based test for clause-embedding predicates: (53)and (54)for *believe* and (55) and (56) for *want*.

(53)

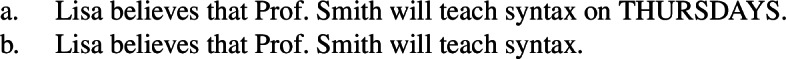

(54)

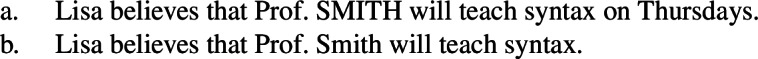

(55)

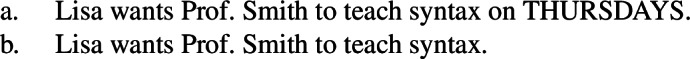

(56)
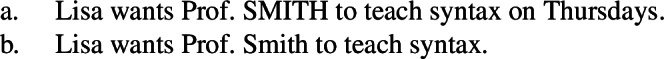

In the case of *believe*, all three consultants judged both inferences to be entailments. Therefore, both variants of the inference-based test would (correctly) conclude that there is no evidence for *believe* being focus-sensitive for any of the three consultants.

Meanwhile, in the case of *want*, only Consultant 2 judged (55)to be a non-entailment, commenting that it could be that Lisa gets Prof. Smith whether she likes it or not. All three consultants judged (56)to be an entailment. Therefore, both variants would classify *want* as focus-sensitive for only one out of our three consultants. That is, if the researcher only applied the inference-based test, they would conclude that there is no evidence for *want* being focus-sensitive for those two consultants who judged (55)to be an entailment.

However, when we further presented these two consultants with the following context (57), which invalidates (55)and aligns with Consultant 2’s comments, both consultants judged (55a) true in this context and judged (55b) and (56a) not true. Therefore, the inference (55)is not in fact an entailment for these two consultants, despite their initial judgments reporting otherwise. In this case, applying only the inference-based test would lead to a false negative. Such an error can be avoided by our proposed test, because the second step of applying the truth-based test would show that (55a) and (56a) have different truth values in the invalidating context (57).

(57) Lisa is indifferent as to who will teach syntax, but she hopes it will be on Thursday. She learns that Prof. Smith will teach it.

The results of our initial investigation suggest that the inference-based test is challenging, at least for non-linguist consultants. In particular, a consultant may fail to consider the relevant invalidating contexts and consequently wrongly judge a non-entailment to be an entailment. When this happens, only applying the inference-based test will yield a false negative, i.e., fail to detect the focus-sensitivity of a predicate, due to a performance error by the consultant.

In general, the inference-based test is likely to involve such performance errors. Indeed, a researcher may collect inference data from multiple consultants in case they suspect performance errors and discover variability in judgments—certain consultants may judge an inference to go through while other consultants may judge that the same pair of examples do not license an inference. Crucially, however, the test by itself does not allow the researcher to tease apart whether such variability arises due to genuine disagreements about the focus-sensitivity status or there are performance errors. In this sense, using the inference-based test alone fails to achieve Tonhauser and Matthewson’s (2016) **stability** criterion, (11), i.e., inclusion of information about factors that may lead to variation in speaker judgments.

The combined method we have presented in Sect. 3 mitigates this issue. In the combined method, inference is used as a way to elicit invalidating contexts, which are then used as contexts against which the truth-based test is conducted. In this setup, even if there is (superficial) variation among participants in the inference judgments, as long as one of the consultants finds an invalidating context, that context can be used for other consultants in the truth-based test. This allows us to reliably determine whether the variation in inference is due to (in)ability to notice an invalidating context or due to a genuine individual variation regarding focus-sensitivity. If the initial inference judgment is due to inability to notice an invalidating context, those consultants will judge the relevant conclusion sentences as false in the truth-based test, if they are explicitly presented with an invalidating context. Conversely, if the initial inference judgment is due to genuine variation, those consultants will judge the relevant conclusion sentences as true even under the (putative) invalidating context elicited by another consultant.^14^

## Remaining issue for our two-step method: potential false negatives

So far, since Sect. 5, we have identified issues with using alternative methods to determine focus-sensitivity of clause-embedding predicates, i.e., the truth-based test alone, the coherence-based test, and the inference-based test alone. We have concluded that the proposed method is more advantageous than these alternatives in light of the desiderata we introduced. This, however, does not mean that the proposed method is absolutely problem-free. We will show below that the definition of focus-sensitivity does not logically entail the relevant properties tested by our proposed method. That is, it is logically possible that our proposed test yields false negatives (of a different type from those discussed before). We speculate that the possibility of such false negatives will be eliminated in practice if we assume two semantic universals. But we acknowledge that, ultimately, the validity of these semantic universals will be left for future empirical investigation. Overall, the goal of the section is to make explicit the empirical assumptions required for the proposed test to operate without false negatives. That is, we aim to maximize the **transparency** of our test.

The issue of potential false negatives we demonstrate in the following applies to both the inference-based part and the truth-based part of the proposed method. We discuss the issue by considering two hypothetical focus-sensitive predicates $B'$ and $B''$, which turn out to be incorrectly classified as non-focus-sensitive by the truth-based part of the test and by the inference-based part of the test, respectively. The issue will be avoided if $B'$ and $B''$ are non-existent in natural language. We will make the assumption explicit in the form of conjectured semantic universals that predict $B'$ and $B''$ as non-existent in natural language.

We first consider the truth-based test. Recall the definition of a focus-sensitive clause-embedding predicate (2), repeated in (58).

(58)

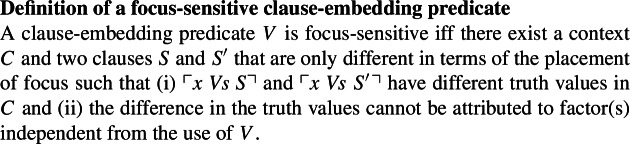

The truth-based part of the test compares the truth values of the following sentences in a context *C* and classifies a predicate as focus-sensitive if the two sentences have different truth values in *C* (and it fails to be conclusive if the truth values are the same).

(59)
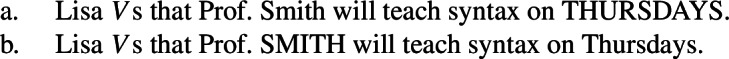

As discussed before, a major limitation of the truth-based test is that there might not be any fully general recipe for constructing relevant contexts. For now we will set this limitation aside, and we will ask whether for any focus-sensitive predicate, the test can in principle correctly identify it as focus-sensitive. That is, if a predicate is focus-sensitive according to the definition in (58), is there always a context *C* such that the two sentences in (59)have different truth values in *C*? It turns out that the answer is no. Consider the following predicate $B'$, defined to be exactly the same as *believe*, except that it always returns FALSE when the set of focus alternatives for the embedded clause is the following set: {Prof. Smith will teach syntax on Thursdays, Prof. Smith will teach semantics on Thursdays, Prof. Smith will teach phonology on Thursdays, …}.

(60)

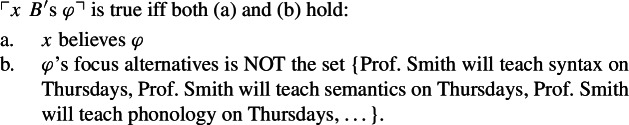

It is easy to verify that $B'$ is indeed focus-sensitive. Suppose that Lisa believes Prof. Smith will teach syntax on Thursdays. According to the definition of $B'$, (61a) is TRUE whereas (61b) is FALSE.

(61)
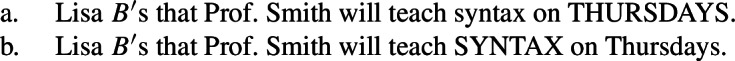

However, since *believe* is not focus-sensitive and $B'$ is equivalent to *believe* in the case of (59a) and (59b), these two sentences will always have the same truth value no matter which context is used and therefore the truth-based test, which is based on the pair (59a) and (59b), can never correctly classify $B'$ as focus-sensitive.

At this point, a natural objection is that the truth-based test should not be tied to the specific sentence pair (59a) and (59b). Rather, the test should be based on any pair of sentences ⌜*x* *Vs* *S*⌝ and $\ulcorner x\ Vs\ S'\urcorner $, where *S* and $S'$ only differ in the placement of focus. Indeed, when the truth-based test is formulated in this way, it can in principle correctly classify any focus-sensitive predicate *V* as focus-sensitive. However, this would lead to another practical problem: not only is there no fully general recipe for constructing the relevant context, there is no general recipe for constructing the relevant pair of sentences, either. That is, in order to correctly handle cases like $B'$, the truth-based test needs to be formulated in a way that makes it even more difficult to be applied in a principled and uniform way.

But is such a formulation really necessary? That is, do we really need to worry about cases like $B'$? The definition of $B'$ is very ad hoc. It only exhibits focus-sensitivity for a specific part of a specific embedded clause. This makes the meaning of $B'$ so unnatural that it is hard to imagine that it would actually be attested in a natural language. Therefore, even though cases like $B'$ can technically be a problem for the formulation of the truth-based test based on a specific sentence pair (59a) and (59b), we suspect that they are not attested in natural languages and therefore in practice we can safely ignore this problem.

More generally, the discussion above shows that the definition of focus-sensitivity in (58), i.e., a predicate (in some context) yields different truth values for *some* pair of embedded clauses, does not logically entail the property assessed by the truth-based test, i.e., that the predicate (in some context) yields different truth values for the particular pair (59) used in our truth-based test. To fill this logical gap, we conjecture the following semantic universal for focus-sensitive predicates in natural languages (62). The intuition behind it is that a focus-sensitive predicate attested in natural language should be focus-sensitive in a uniform way, in that it should exhibit focus-sensitivity regardless of which particular clause it embeds.

(62)
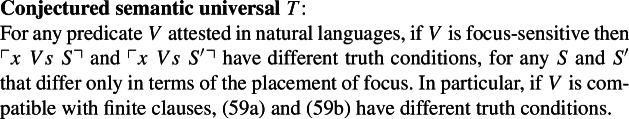

This conjectured universal, if correct, will ensure that the truth-based test based on a specific pair of sentences (59a) and (59b) can in principle correctly classify any focus-sensitive predicate attested in natural languages as focus-sensitive.

Now we return to the inference-based part of the test. Recall that its general structure is as follows (63).

(63)
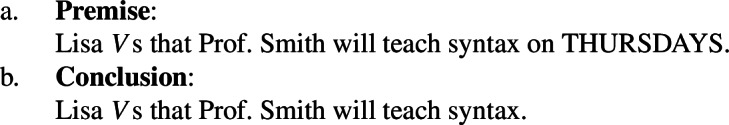

Consultants are asked whether (63a) entails (63b). If they all judge this inference to be an entailment, then the researcher concludes that there is no evidence for *V* being focus-sensitive for any consultant.

However, technically, a predicate can be focus-sensitive and still validate the inference from (63a) to (63b). For instance, consider the following predicate $B''$, which is defined to be almost exactly the same as *believe*, except that it will return FALSE when the set of focus alternatives of the embedded clause is {Prof. Smith will teach syntax on Mondays, Prof. Smith will teach syntax on Tuesdays, …}.

(64)

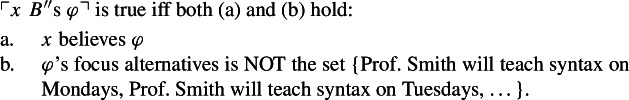

Given the definition of $B''$, the premise (65a) is always FALSE (since the focus alternatives of the embedded clause match the special clause in the definition). Therefore (65a) trivially entails (65b). Consequently, our proposed test would conclude that there is no evidence for $B''$ being focus-sensitive.

(65)
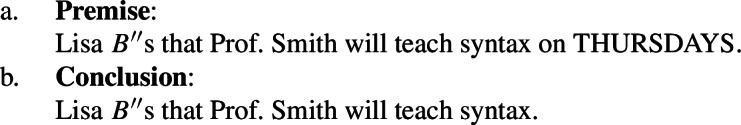

However, in the following context (66), (67)is TRUE because $B''$ is equivalent to *believe* in this sentence, whereas (65a) is FALSE (since it is always FALSE given how $B''$ is defined). Therefore, $B''$ is in fact focus-sensitive, but our test will yield a false negative because (65)is an entailment.

(66) **Context**: Lisa believes that Prof. Smith will teach syntax on Thursdays.

(67) Lisa $B''$s that Prof. SMITH will teach syntax on Thursdays.

The discussion above shows that the definition of focus-sensitivity in (58) also does not logically entail the property assessed by our inference-based test, i.e., the predicate invalidates the inference in (63). Again, to what extent should we be worried about this? We observe that in this case focus-sensitivity again comes from a highly idiosyncratic use of focus alternatives: similar to $B'$, $B''$ is doing something special only to some particular sets of focus alternatives, whereas canonical focus-sensitive predicates such as *hope* and *be surprised* use focus alternatives in uniform ways. This suggests that while technically predicates like $B''$ can lead to false negatives, their focus-sensitive meanings may be too artificial to actually be realized lexically, and therefore in practice they may not present a real challenge to the inference-based test.

More generally, in parallel with (62), we tentatively propose the following semantic universal (68).^15^

(68)
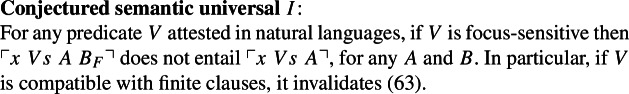

If this conjecture is true, then predicates like $B''$ do not actually exist in natural languages, and we do not need to worry about this type of false negatives.

Note that $B''$ is also ruled out by our conjectured universal *T* (62). Nevertheless, we emphasize that the conjectured universal *I* is independently needed, because not all predicates that it rules out can be ruled out by *T*. For instance, consider the following definition of a predicate *P* suggested by a reviewer:

(69)
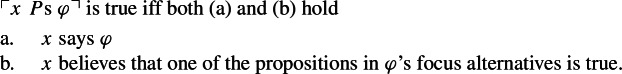

Given this definition, the truth conditions of the premise and conclusion used in the inference-based test would be the conjunction of the corresponding (a)- and (b)-clauses.

(70)

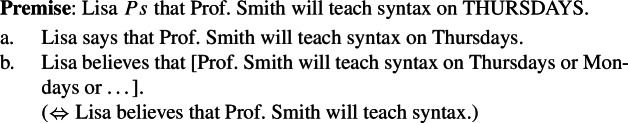

(71)
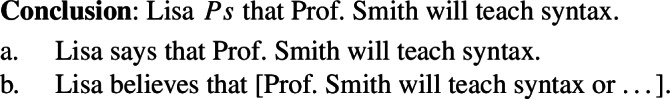

Given that (70a) entails (71a) and (70b) entails (71b), (70)entails (71).^16^ Furthermore, we can verify that *P* is focus-sensitive: in the following context (72), (70)is TRUE but (73)is not.

(72) **Context**: Lisa believes that Prof. Smith will teach syntax on Tuesdays. (And given the background assumption that there is exactly one syntax class on exactly one day of the week, Lisa does not believe that anyone will teach syntax on Thursdays.) But she says that Prof. Smith will teach syntax on Thursdays.

(73) Lisa *P*s that Prof. SMITH will teach syntax on Thursdays.

Therefore, *P* is a focus-sensitive predicate and its meaning is (presumably) not as intuitively implausible as $B''$. In particular, since the definition of *P* does not make reference to particular sets of focus alternatives or particular position of focus placement, it will not be ruled out by the universal *T*. However, it is still ruled out by our conjectured semantic universal *I* above. Indeed, as far as we know, there are no attested instances of *P* in natural languages.

Of course, it is ultimately an empirical question whether the conjectured semantic universals *T* (62) and *I* (68) in fact hold. If they do, it would also be interesting to see whether these universals can be theoretically derived based on some more fundamental assumptions about focus-sensitivity and lexicalization. And if they do not, it would be important to examine the counterexamples, which hopefully would lead to a more accurate universal that characterizes the range of attested focus-sensitive meanings. Even though addressing these questions is beyond the scope of the current paper, we note that all the predicates that have been suggested to be focus-sensitive that we know of are consistent with (62) and (68), which lends some initial support to the conjectures.^17^

Where does the above discussion lead us with respect to the comparison of our proposal with the alternatives? The bottom line is that both the inference-based test and the truth-based test are not absolutely/logically immune to false negatives, and thus are not completely transparent in Tonhauser and Matthewson’s (2016) sense, i.e., it is not fully clear how the test supports a given empirical statement about the focus-sensitivity of a predicate (since it further relies on conjectured universals whose correctness have not be proven). This makes our proposed method—which relies on both the inference-based and the truth-based test—not completely transparent. At the same time, this is an issue the method shares with alternative methods that utilize the inference-based test alone and truth-based test alone. In this sense, this feature does not make our proposed method less advantageous than these alternative methods in terms of transparency.

## Summary and outlook

A number of analyses of the syntactic and semantic behaviours of clause-embedding predicates make crucial use of the property of focus-sensitivity (e.g., Villalta 2008; Romero 2015; Uegaki and Sudo 2019; Wehbe and Flor 2022). However, although the analytical intuition behind this property—that the predicate is sensitive to the focus structure of its complement—is relatively straightforward, there are challenges associated with the construction of a concrete empirical test for focus-sensitivity that can be applied to any arbitrary predicate in a cross-linguistic setting.

In this paper, we have offered the practical methodological recommendation of combining an inference-based test and a truth-based test for detecting focus-sensitivity. Combining the inference- and truth-based tests by using the inference-based test as an initial step to elicit relevant contexts for the truth-based test can make the two tests complement each other. On the one hand, the inference-based test can make the combined test generally applicable. On the other, the truth-based test can yield more stable and replicable results, and the correctness of the results does not hinge on whether the predicate is upward entailing.

We have assessed the relative advantage of this recommended method in relation to three types of empirical methods for detecting focus-sensitivity: the truth-based test only, the coherence-based test, and the inference-based test only, and discussed their advantages and limitations. The truth-based test presupposes the ability to construct suitable contexts against which the truth value of target sentences can be judged, which either is not possible when the researcher is yet to uncover the precise lexical semantics of a target predicate or not possible at all without contradiction. The coherence-based test suffers from a confound arising from the ambivalent nature of the denial expression used in the test. The outcome of the test varies depending on whether the denial expression targets falsity or infelicity, which makes it difficult to formulate the test in a cross-linguistically general manner. The inference-based test is free from these challenges, and can constitute a test format that is general with respect to both predicates and languages. However, using the inference-based test alone faces further challenges associated with a practical difficulty in detecting inferences/non-inferences as well as with non-upward-entailing predicates.

By examining the methodological challenges associated with testing for focus-sensitivity, we hope to have contributed to the ongoing discussion on the methodology of controlled semantic data collection in the cross-linguistic context (Matthewson 2004; Tonhauser and Matthewson 2016). In particular, our contribution lies in setting one of the precedents for an in-depth *phenomenon-specific* investigation of the semantic data collection methodology. We believe such phenomenon-specific investigations are particularly relevant as the field increasingly relies on cross-linguistic observations in a diverse set of phenomena, where the quality of data assessment methods is crucially important.^18^

We would like to conclude this paper by comparing the general methodological position that underlies our proposal with the methodological positions that are most commonly taken in semantic fieldwork and in experimental semantics, respectively. We believe our position is relatively unique in that it incorporates practices from experimental semantics to semantic fieldwork. Although elicitation of judgments concerning entailment is not commonly used in semantic fieldwork (cf. Matthewson 2004), it is commonly used in current experimental semantics and more generally for collecting training and test data used in computational linguistics (e.g., Dagan et al. 2006; White and Rawlins 2018; Degen and Tonhauser 2022). In this sense, our position is in line with a large body of work in experimental semantics. At the same time, however, our methodology drastically differs from standard practices in experimental semantics and is closer to fieldwork semantics in emphasizing the importance of *qualitative* judgments. In our methodology, the inference-based test is an initial prompt that facilitates a discussion between the researcher and the consultant about the precise nature of the consultant’s judgment about inference (or lack thereof). If the consultant judges the inference to hold, the researcher may ask the consultant whether they have considered certain specific contexts. If the consultant judges the inference not to hold, the researcher may ask in which concrete contexts the inference breaks. Such qualitative judgments are typically not part of the data gathered in experimental semantics, and in this respect, our methodology aligns more with fieldwork semantics.

Our general methodological choice is rooted in our belief that the traditional semantic notion of inference/entailment is a useful primitive notion that can be insightfully investigated in cross-linguistic data collection (in line with experimental semantics). At the same time, it would be counterproductive to treat native speaker consultants as “subjects” who only provide raw judgments because—we believe—they can provide qualitative data that are theoretically informative about what underlies those judgments (contra standard practice in experimental semantics; and also contra some claims in the fieldwork semantics literature; see Louie 2015). We hope to have shown that cross-linguistic investigation of focus-sensitivity is a domain where this hybrid methodological position is particularly suitable.

## Footnotes

1 Our discussion of focus is independent of the description of how focus is realized within a given language and questions like whether association with focus is conventional or non-conventional (Beaver and Clark 2007). Some terminological choices we make are as follows. We sometimes call two strings of the form ⌜…A B_*F*_…⌝ and ⌜…A_*F*_ B…⌝, which only differ in the position of focus, the same sentence, and sometimes we call them two different sentences. We use the terms “clause-embedding” and “attitude” verb or predicate interchangeably, and assume that verbs are a subset of predicates, the latter including, e.g., adjectival forms like *be glad* or *be surprised*.

2 The definition roughly corresponds to Harner’s (2016, p. 28) definition of *semantic focus-sensitivity for attitude predicates*.

3 Here we adopt a *conservative* formulation of the test, where a researcher only concludes that they fail to find evidence for a predicate being focus-sensitive. Alternatively, one can also adopt a *radical* formulation of the test, where a researcher draws a stronger conclusion that a predicate is not focus-sensitive when they fail to find evidence for focus-sensitivity. Later, when we evaluate our test and various alternative approaches, we say that a test yields a false negative for a predicate iff the predicate is focus-sensitive and yet the researcher applying the test fails to find evidence that allows them to conclude that it is so. If one instead adopts the radical formulation of a test, then correspondingly a false negative would be one where the predicate is focus-sensitive but the researcher concludes that it is not. For the purpose of this paper, there is no crucial difference between these two formulations.

4 More precisely, since the relevant inference will be evaluated against a pre-specified broad context, the relevant notion of entailment is a contextual entailment and an invalidating context is a refinement of the broad context. For brevity, throughout this paper, we will simply use the term *entailment*.

5 This test was inspired by the discussion in Dretske (1972). We thank Felix Frühauf (p.c.) for drawing our attention to Dretske’s discussion.

6 The situation might be more difficult in a monolingual fieldwork setting.

7 Villalta says “not true” rather than “false”, presumably to leave room for the possibility that a target sentence may also come out as infelicitous. Harner (2016) draws a distinction between semantic and pragmatic focus-sensitivity, depending on whether focus leads to a difference between truth and falsity (semantic) or felicity and infelicity (pragmatic). A truth-based test can in principle be formulated in a way that takes this difference into account, so long as consultants can reliably distinguish between falsity and infelicity (though see Matthewson 2004; Deal 2015).

8 In (14c-i), the constituent “on Tuesdays and Thursdays” is also where focus projects from, and where main sentential stress is expected to be realized in broad(er) focus contexts (Selkirk 1996, a.o.). For uncontextualized realizations of (14c-i), this makes it difficult to tell when “on Tuesdays and Thursdays” is under narrow focus vs. when it is part of a larger focused constituent. Villalta does not address this confound, and we will not fully do so either. In practice, the contexts, the target sentences and possibly other task-related information involved in the tests presented in this paper would all seem to bias consultants into narrow focus construals. The context in (14), for example, explicitly contrasts two sets of days, which evokes narrow focus alternatives to sentences with sentential stress on “on Tuesdays and Thursdays”.

9 In principle, one could include only the first half of the context, for which there is indeed a general recipe, i.e., ‘not P which day these classes take place’ (modulo the need for paraphrases when P does not embed questions). However, this would make the dialogue less natural, and does not avoid the limitations we will discuss below.

10 In fact, a different test based on this idea would be to directly test for the consistency of sentences such as “Ann wants Lisa to teach syntax on THURSDAYS, but she doesn’t want LISA to teach syntax on Thursdays”. Such a test also faces the concerns that the sentence may not sound very natural and that negation might be used meta-linguistically. Additionally, for a neg-raising predicate *P*, ‘Ann Ps *φ* and Ann does not P $\varphi '$’ (where *φ* and $\varphi '$ only differ in the placement of narrow focus) is likely interpreted as $P\varphi \wedge P\neg \varphi '$, which is a stronger claim than the intended $P\varphi \wedge \neg P\varphi '$. Therefore, it is possible that the intended interpretation $P\varphi \wedge \neg P\varphi '$ is consistent but the strengthened $P\varphi \wedge P\neg \varphi '$ is not, and the consultant’s judgment is for the strengthened interpretation because it is more salient.

11 Note that this is a place where $D''$ may be different from English *deny*. In the case of *deny* (at least for some speakers) Premise 2 (i.e., ‘Lisa denies that Prof. SMITH will teach syntax on Thursdays’) strongly implies that Lisa agrees/believes that syntax will be taught on Thursdays. If this implication is an entailment, then Premise 2 is not true in scenario (44) and hence (44) will not be an invalidating context.

12 In light of this, one might be tempted to reformulate the 2-inference variant so that it classifies the predicate as focus-sensitive when neither inference is an entailment. This reformulation would correctly classify $D''$ as focus-sensitive. However, given the definition of $D'$ in (38), we can verify that neither (46a) nor (46b) entails (47)due to the invalidating context (39). Therefore this reformulation would incorrectly classify $D'$ as focus-sensitive.

13 They reported ‘British English’, ‘RP standard English’ and ‘standard English’ as their respective language backgrounds, and were living in Edinburgh at the time of elicitation.

14 Of course, there is in principle the possibility that *none* of the consultants detect a non-entailment even if the predicate is in fact focus-sensitive. This challenge, however, can be mitigated by increasing the number of samples/consultants, in a way similar to the process of increasing the statistical power in psycholinguistic experiments. The nature of this challenge is inherently different from that of using the inference-based test alone. If a researcher uses the inference-based test alone, the risk of conflating performance errors and genuine variation cannot be reduced even if they increase the number of participants.

15 Note that this conjecture is uni-directional. It does not say that all predicates invalidating (63) are focus-sensitive.

16 Here we abstract away from the question of what the relevant focus alternatives are for (71). As long as the embedded clause itself is assumed to be a member, the entailment will hold.

17 We acknowledge that even though there is some parallel between (62) and (68) and, to the best of our knowledge, that they are both correct, they may not be perfectly comparable. As discussed above, the intuition behind (62) is rather straightforward: if a predicate is focus-sensitive, it should exhibit focus-sensitivity regardless of which clause it embeds. It is unclear how to similarly motivate (68).

18 We thank a reviewer for encouraging us to emphasize this point.
